# Relations of admiration and adoration with other emotions and well-being

**DOI:** 10.1186/s13612-014-0014-7

**Published:** 2014-08-19

**Authors:** Ines Schindler

**Affiliations:** Free University Berlin, Cluster (Languages of Emotion), Habelschwerdter Allee 45, 14195 Berlin, Germany

**Keywords:** Admiration, Adoration, Reverence, Worship, Gratitude, Inspiration, Fascination, Envy, Psychological well-being, Life satisfaction

## Abstract

**Background:**

Admiration and adoration (also referred to as reverence or worship) have 2 received little empirical attention, although the two emotions theoretically have been related to individual and collective well-being. This research tested for associations of dispositional admiration and adoration with dimensions of psychological well-being and life satisfaction.

**Methods:**

We developed a new measure of dispositional admiration and adoration and employed it in a questionnaire study with 342 participants. Additional measures included various emotion dispositions and dimensions of well-being.

**Results:**

While admiration was linked to greater levels of personal growth and adoration to greater levels of purpose in life, the two emotions were unrelated to environmental mastery, self-acceptance, and life satisfaction. A multiple-step multiple mediator model revealed that counteractive positive and negative indirect effects of admiration and adoration on mastery, self-acceptance and life satisfaction were hidden beneath the nonsignificant total effects. Specifically, there were positive indirect effects of admiration and adoration via inspiration and gratitude and negative indirect effects via fascination and envy on well-being.

**Conclusions:**

Taken together, the findings suggest that admiration and adoration bind people to ideals irrespective of their ability to move closer to them, thereby providing a potential source of satisfaction as well as frustration.

## Background

It is easy to find (self-help) publications advancing that admiration, adoration, or reverence benefit people (e.g., Gallozzi 2009; Perticone 2007; Rattner and Danzer 2006; Woodruff 2001). According to Rattner and Danzer (2006) “adoring and admiring is one of the most important means for the development and growth of a human’s personality” (p. 27; author, Trans.). Gallozzi (2009) stated that “besides being a valuable teacher, admiration is a source of happiness” (“We don’t grow up,” para. 8). Such statements are supported by pointing to famous people like Friedrich Nietzsche, Rainer Maria Rilke (cf. Rattner and Danzer 2006), or Thomas Mann (cf. Gallozzi 2009), who have started out as admirers and even worshippers of other famous people.

In contrast to such biographical evidence, to the best of my knowledge, there is no empirical study demonstrating associations of emotions like admiration, adoration, reverence, or worship with personal growth, happiness, or well-being in a broader sample. Has psychology overlooked admiration and adoration as important contributors to well-being? Or are associations of these emotions with well-being not as straightforward as suggested above?

We^a^ conducted the present research to fill this gap in the literature. Specifically, we examined whether and how dispositional admiration and adoration were related to well-being. To accomplish this, we first needed to gain more insight into admiration and what has variously been labeled adoration, reverence, worship, or veneration (I will use the term *adoration* to denote the core meaning of this word cluster). There are rather few psychological publications considering these emotions (but see Algoe and Haidt 2009; de Rivera 1977; Haidt 2003a, b; McDougall 1921; Ortony, Clore, and Collins 1988; Plutchik 1980; Smith 2000; van de Ven, Zeelenberg, and Pieters 2011, 2012). Therefore, Schindler, Zink, Windrich, and Menninghaus (2013) have provided a theoretical account of the characteristics of and differences between admiration and adoration, which I will introduce in the next section. Based on Schindler et al. (2013), we developed a questionnaire assessing dispositional admiration and adoration to elucidate associations of the two emotions with well-being. As a prerequisite for addressing this link, I will first present evidence supporting the reliability and validity of the new measure. Subsequently, I will uncover how admiration and adoration are related to well-being indicators.

### Admiration and adoration

Admiration and adoration are positive emotions in response to an outstanding person or object. Theoretically (Schindler et al. 2013), these emotions should serve to keep a person’s ideals and values accessible as guides for behavior and also contribute to the adoption and internalization of ideals, values, and goals. However, the underlying processes are different.

As detailed in Schindler et al. (2013), admiration is elicited by outstanding role models who represent specific ideals or values. The excellence of such models, at least in principle, can be understood, matched, and even surpassed by others. Admired others can encourage people who aspire to grow by showing that it is possible to actualize ideals. The action tendencies associated with admiration are to uphold and honor ideals. The admiring individual seeks to praise and affiliate with the other as well as to emulate the other’s conduct (e.g., Algoe and Haidt 2009; Aquino, McFerran, and Laven 2011; Haidt 2003a). Thus, the primary function of admiration is to enhance the individual’s agency in striving for ideals.

Adoration is elicited by excellence that cannot be fully understood or attained by anyone else: Adherents perceive adored others as superhuman or sacred. The other embodies an ideal state of being that is forever out of ordinary people’s reach, but that these people would like to share in and benefit from. The only way to accomplish this is to please or to unite with the adored other. The central action tendencies of adoration are to seek to establish a relationship with the other (if only in thought), to make him or her a part of one’s identity, and to adopt the ideals, values, and meanings which are transferred by the other (cf. Durkheim 1915/2008; Plutchik 1980; Weber 1956/1978). Rather than being a role model, the adored other serves as a meaning maker and benefactor able to unite adherents under his or her guidance. Accordingly, the primary function of adoration is to create and maintain social cohesion.

#### Relations of admiration and adoration with other positive emotions

Given admiration’s and adoration’s positive valence, one might expect that individuals with a disposition to experience positive affect also tend to feel these emotions. However, positive affect is not a unitary construct, but specific positive emotions serve different functions (e.g., Griskevicius, Shiota, and Neufeld 2010; Lazarus 1991; Plutchik 1980). While global positive affect can be conceived of as indicating goal progress or need fulfillment (cf. Carver and Scheier 1998), it is important to recognize that this characterization matches only some positive emotions like joy, happiness, contentment, or pride (cf. Fredrickson 1998; Lazarus 1991). Other positive emotions, most clearly love, help develop and maintain relationships (cf. Fredrickson 1998; Lazarus 1991). Still other emotions, like interest or inspiration, are linked to setting goals and standards or to taking on challenging tasks (Fredrickson 1998; Thrash and Elliot 2004; Vittersø and Søholt 2011; Vittersø, Søholt, Hetland, Thoresen, and Røysamb 2010).

Based on their appraisal patterns and functions (Schindler et al. 2013), admiration and adoration can be connected to the latter two sorts of positive emotions, but not to positive emotions resulting from goal progress. First, admiration and adoration express valuation of others and, thus, are linked to the positive affective disposition to appreciate goodness in others. Accordingly, the two emotions have been considered as members of an emotion family labeled as *appreciation* or *liking* emotions (cf. Peterson and Seligman 2004; Ortony et al. 1988) and *other-praising* emotions (Algoe and Haidt 2009; Haidt 2003b). The present study included love and gratitude as two other members of this emotion family (cf. Haidt 2003b; Lazarus 1991; McCullough, Kilpatrick, Emmons, and Larson 2001; Ortony et al. 1988).

Second, admiration and adoration connect to the disposition to respond to cognitively challenging stimuli with positive affect. This challenge is experienced as a need to accommodate one’s knowledge structures. Thus, admiration and adoration should relate to other emotions resulting from the transcendence of one’s prior knowledge and experience. The connection with awe (Keltner and Haidt 2003) is most evident, as awe is closely related to adoration (cf. Schindler et al. 2013): Closeness to adored others elicits awe. However, awe can be experienced without adoration. Other relevant emotions included in the present study are inspiration (Thrash and Elliot 2003, 2004) and fascination (Kaplan 1995; Lüdtke, Jäkel, and Ordonez Acuna 2013).

#### Relations of admiration and adoration with negative emotions

In contrast to prototypical positive emotions, admiration and adoration have been discussed as compound emotions that include negative components. For instance, McDougall (1921) described admiration as a compound of the primary emotions wonder and negative self-feeling and reverence as a compound of wonder, negative self-feeling, fear, and tenderness. According to Schindler et al. (2013), admiration and adoration do not necessarily involve negative feelings, but the two emotions can occur in connection with or turn into negative emotions, especially when attention gets focused on the self. In relation to an admired or adored other, people may perceive themselves as lacking important qualities or skills, inferior to the other, or dependent on the other’s benevolence, which may give rise to feelings of sadness, fear, or shame.

Admiration further has been discussed in relation to envy, as both emotions can occur in upward social comparison situations (Cohen-Charash 2009; de Rivera 1977; Smith 2000; van de Ven et al. 2011, 2012). In a given situation, admiration and envy are incompatible. Admiration rather than envy results when the other’s superiority is deserved and does not reflect badly upon oneself (Feather, McKee, and Bekker 2011; van de Ven et al. 2012). Nevertheless, there may be a positive association between admiration and envy when we move to the dispositional level. The person who considers an ideal as important can admire others who are not too similar, represent the ideal, and deserve it. At the same time, they can envy similar others who represent the ideal without deserving it. Consider, for instance, an employee who values competence and career and, thus, admires the outstanding achievements of her boss who has twenty more years of experience on the job and envies her equally experienced but more successful colleague.

In contrast to admiration, adoration has not been related to envy. First, adored others cannot serve as realistic standards for comparison (Schindler et al. 2013). Second, if envy among the adherents of an adored other were to increase together with adoration, this would undermine adoration’s community-binding function. In line with this reasoning, Paris (2010) described envy as a socially stigmatized emotion which isolates individuals. Schurtz et al. (2012) suggested that envy undermines social hierarchies while awe stabilizes them.

### Well-being

The central question of this paper was whether admiration and adoration are linked to well-being. Psychologists have conceptualized well-being and its constituents within different research traditions and perspectives. A central distinction is the one between hedonic well-being and eudaimonic well-being (cf. Kashdan, Biswas-Diener, and King 2008; Ryan and Deci 2001; Waterman 2008). In the hedonic tradition, well-being is defined in terms of pleasure and happiness as *subjective well-being* (SWB; Diener 1984; Diener, Suh, Lucas, and Smith 1999). In the eudaimonic tradition, well-being is defined in terms of actualizing one’s full potential or true nature. Researchers have posited somewhat different conceptualizations of eudaimonic well-being (see Kashdan et al. 2008; Ryan and Deci 2001; Waterman 2008, for overviews). In this paper, I will focus on *psychological well-being* (PWB; Ryff 1989; Ryff and Singer 2008) as one construct in the eudaimonic tradition. The current study included measures of both subjective and psychological well-being to cover the entire range of well-being indicators that could relate to admiration and adoration.

SWB reflects people’s evaluations of their lives based on their own standards. It includes life satisfaction as a cognitive component and high positive and low negative affect as an affective component (Diener 1984; Diener et al. 1999). As my focus was on connections of different positive and negative emotions with well-being, I included only the cognitive component of SWB in the form of global life satisfaction. However, assessments of life satisfaction merely tell us whether someone is satisfied with his or her life, not why this individual is satisfied. In the previous sections, I have identified two pathways through which admiration and adoration link to life satisfaction, namely, by promoting individual growth and by offering a framework of meaning. To study such pathways, I also examined dimensions of PWB.

The employed measure of PWB comprises six key dimensions (Ryff 1989; Ryff and Singer 2008): autonomy, positive relations with others, personal growth, purpose in life, environmental mastery, and self-acceptance. Although the SWB and eudaimonic well-being (including PWB) approaches originated from different research streams, they are conceptually and empirically related. For instance, Waterman (2008) argued that pleasure (as one aspect of SWB) results when someone is getting what he or she wants and that dimensions of eudaimonic well-being represent the things that people may want. Therefore, the attainment of eudaimonic well-being is sufficient, but not necessary, to create SWB. Other studies have demonstrated that measures of SWB and PWB form separate but highly correlated factors (Keyes, Shmotkin, and Ryff 2002; Linley, Maltby, Wood, Osborne, and Hurling 2009). Among the PWB dimensions, self-acceptance and environmental mastery consistently showed stronger associations with SWB variables than the remaining four dimensions (Keyes et al. 2002; Ryff and Keyes 1995).

It also has been suggested that some dimensions of eudaimonic well-being make little, if any, contribution to SWB. Rather, it is possible to separate between happiness or satisfaction and meaning, purpose, or growth as two ingredients of a life well lived (McGregor and Little 1998; Vittersø and Søholt 2011; Vittersø et al. 2010). In support of this view, there is research showing that meaning in life or growth are weakly related, unrelated, or even negatively related to measures of SWB (e.g., Bauer, McAdams, and Sakaeda 2005; Delle Fave, Brdar, Freire, Vella-Brodrick, and Wissing 2011; Keyes 2000; McGregor and Little 1998). Thus, some PWB dimensions, and in particular self-acceptance and environmental mastery, can be expected to serve as pathways to life satisfaction, while others show weaker or no relations with life satisfaction. Especially purpose in life and personal growth have been considered as central to meaning rather than happiness (Ryff and Singer 1998; see also McGregor and Little 1998).

#### Relations of admiration and adoration with dimensions of psychological well-being

Theoretically, it is possible to say that admiration motivates to grow by putting oneself in the place of the person who upholds an ideal (Schindler et al. 2013). Adoration motivates to find purpose by embracing the meanings and values conveyed by an ideal person or being. This characterization provides a direct link to the PWB dimensions personal growth and purpose in life. Specifically, dispositional admiration should show a positive relation with personal growth and adoration should be linked to having purpose in life.

#### Relations of admiration and adoration with life satisfaction

If one were to reduce admiration and adoration to their positive affective valence, it would be straightforward to conclude that these emotions should be positively related to life satisfaction. After all, life satisfaction and positive affect both are indicators of SWB (cf. Diener 1984; Diener et al. 1999). Moreover, the PWB dimensions personal growth and purpose in life are positively correlated with life satisfaction (Keyes et al. 2002; Ryff and Keyes 1995). If the hypothesized associations of admiration with personal growth and of adoration with purpose in life exist, the two PWB dimensions could function as mediators of a positive relation of admiration and adoration with life satisfaction. Recent evidence by Rudd, Vohs, and Aaker (2012) showing that inducing awe can increase momentary life satisfaction also suggests that the closely related emotion adoration is linked to greater satisfaction.

However, if admiration and adoration also were related to negative affect, this may undermine positive associations with life satisfaction. Furthermore, the PWB dimensions personal growth and purpose in life are less important predictors of life satisfaction than other PWB dimensions. If admiration and adoration showed little or no relation with these more important dimensions, this would also suggest a rather small or no relation of admiration and adoration with life satisfaction.

### Summary of research questions and hypotheses

The developed hypotheses pertain to the two goals of this study. First, in order to validate a new measure of dispositional admiration and adoration, their relations with other emotions and selected PWB dimensions were considered. Admiration and adoration should demonstrate discriminant validity from positive emotions that respond to goal progress, that is, show only small or no positive correlations with joy and pride (Hypothesis 1). In terms of convergent validity, admiration and adoration were hypothesized to overlap with emotions that result from appreciating others and needing to accommodate one’s knowledge structures. Specifically, I expected that admiration and adoration are positively correlated with love and gratitude (Hypothesis 2) as well as awe, inspiration, and fascination (Hypothesis 3).

Admiration and adoration further should be discriminable from other positive emotions by their distinct relations with other positive and negative emotions. Admiration and adoration were hypothesized to show no or even positive rather than negative associations with sadness, fear, shame, and envy (Hypothesis 4). Dispositional envy further was predicted to relate positively to admiration but not or negatively to adoration (Hypothesis 5).

Finally, admiration and adoration have been linked to two of the six PWB dimensions. I expected admiration to relate positively to personal growth (Hypothesis 6). Adoration should be linked to greater purpose in life (Hypothesis 7).

The major aim of this study was to clarify associations of admiration and adoration with various dimensions of PWB and life satisfaction. As the extant literature does not allow to derive clear-cut predictions for all possible associations, I included this as an open research question: How are admiration and adoration related to the remaining PWB dimensions (other than personal growth and purpose in life) and life satisfaction (Question 1)?

**Figure 1:**
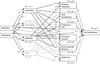
**Multiple-step multiple mediator model linking admiration and adoration to life satisfaction.** Standardized path coefficients are reported. Solid lines show positive associations, dotted lines show negative associations. The covariance between admiration and adoration and residual covariances between mediators were estimated but were omitted from the figure to reduce complexity. ***p < .001. **p < .01. * *p* < .05. + *p* < .10.

Based on the complex relations of admiration and adoration with other positive and negative emotions it further seemed interesting to consider the role of associated emotions in explaining associations with well-being. Specifically, the question was whether relations of admiration and adoration with well-being constructs can be explained by taking relations of admiration and adoration with other emotions into account (Question 2). I specified a multiple-step multiple mediator model (cf. Hayes 2009; Williams and MacKinnon 2008) to address this question. The model included admiration and adoration as predictors, associated positive and negative emotions as mediators in step 1, the six PWB dimensions as mediators in step 2, and life satisfaction as outcome variable (Figure 1). However, this model should *not* be interpreted to depict causal relations. For instance, I do not mean to suggest that dispositional admiration causes dispositional gratitude (rather, the two emotions can result from a common cause). The model merely was intended to determine how admiration, by way of its association with gratitude, related to the PWB dimensions and life satisfaction.

## Method

### Participants and procedure

#### Sample recruitment

Participants were recruited through advertisements on the Berlin underground, postings in online discussion forums, distribution of flyers, and invitations to personal acquaintances. Advertisements directed persons to an online registration portal. During registration, people also provided some initial data. In addition to giving us basic demographic information, they listed persons or things they admire and persons or things they adore. This information helped us select persons to be invited for the main study.

We invited 436 of the 538 registered persons to participate. We focused on people who had nominated at least one admired (*n* = 492, 91.4%) and/or one adored (*n* = 297, 55.2%) person or object. In addition, we invited 30 persons who had not registered online but had previously participated in our research or were personal acquaintances. Finally, we invited some persons who had claimed to experience no or very infrequent admiration and adoration to obtain the full range of scores for the two emotions.

#### Procedure

The 398 individuals who agreed to participate (85.4% of those invited) received a questionnaire containing various questions targeting their emotional experiences, well-being, personality characteristics, values, and goals. This questionnaire took an estimated 60 to 90 minutes to complete. Participants typically received the questionnaire via mail as a paper-and-pencil version along with a return envelope postage paid. Participants received a computerized version of the questionnaire via e-mail if mailing it was not feasible or if the participant preferred this (3.3% of the distributed questionnaires). Participants received a monetary compensation of 10 Euro.

#### Sample description

We received questionnaires of 343 participants (a return rate of 86.2%), including 212 women (61.8%) and 131 men (38.2%) aged between 18 and 73 years, *M* = 34.0 years, *SD* = 12.2. The vast majority of participants were native speakers of German (94.5%), but the sample also included some persons with a different native language who were fluent in German (5.5%). Educational attainment in the sample was diverse but fairly high on average, with 87.7% having obtained the highest possible level of schooling (i.e., qualification for college or university) and 43.1% having graduated from college or university (i.e., bachelor, masters, or doctoral degree or equivalents thereof). At the time of the study, 44.0% of the participants reported that their primary occupation was the pursuit of their (further) education or vocational training, 20.1% of the participants were working full time and 10.5% part time, 12.5% were not in paid employment (including people who were unemployed, on leave, or retired as well as full-time homemakers), and 12.8% indicated some other primary occupation (including self-employment, military or civil service, and nonresponse).

### Measures

All measures were administered in German using 5-point Likert scales (with higher scores indicating greater frequency/agreement). Scale scores were formed by averaging across the respective items (descriptives are reported in Table 1). The first section of the questionnaire after initial questions on demographic information assessed a range of positive emotions (items for different emotions were mixed), including admiration, adoration, joy, love, pride, inspiration, fascination, and awe. Participants indicated how often, in general, they have each emotion (this instruction was used to assess affective disposition rather than affective state).

**Table 1:** **Descriptives, correlations, and partial correlations of study variables**

**Variable**	***M***	***SD***	**1**	**2**	**3**	**4**	**5**	**6**	**7**	**8**	**9**	**10**	**11**	**12**	**13**	**14**	**15**	**16**	**17**	**18**	**19**	**20**
1 Admiration	3.62	0.66				.04	.04	.16**	.19***	.17**	.13*	.02	.08	.08	.20***	-.20***	.07	.18**	.06	-.00	.01	.02
2 Adoration	2.91	1.02	.63***			.02	.06	.14**	.06	.10+	.13*	-.06	-.02	-.03	-.11*	.02	-.02	-.02	.13*	.12*	.04	.06
3 Awe	2.86	0.94	.62***	.71***		.03	-.03	-.10+	.03	.01	.24***	.05	.06	.05	.11+	.03	-.01	-.08	-.02	-.11*	-.05	.00
4 Joy	3.64	0.81	.11+	.10+	.10+																	
5 Pride	3.19	0.75	.08	.09	.05	.69***																
6 Love	3.97	0.82	.24***	.23***	.13*	.47***	.34***															
7 Gratitude	3.93	0.62	.33***	.28***	.26***	.56***	.37***	.39***														
8 Inspiration	3.54	0.74	.31***	.28***	.24***	.31***	.39***	.14**	.28***													
9 Fascination	3.36	0.72	.44***	.48***	.52***	.08	.10+	.16**	.23***	.40***												
10 Sadness	2.15	0.77	.01	-.02	.02	-.64***	-.50***	-.34***	-.34***	-.17**	.04											
11 Fear	2.07	0.65	.14*	.09+	.13*	-.52***	-.47***	-.14*	-.20***	-.09	.18**	.62***										
12 Shame	1.71	0.78	.12*	.06	.10+	-.34***	-.36***	-.14**	-.07	-.08	.13*	.42***	.49***									
13 Envy	1.94	0.62	.26***	.11*	.20***	-.43***	-.41***	-.04	-.28***	-.14**	.14**	.41***	.50***	.30***								
14 Autonomy	3.57	0.58	-.23***	-.11*	-.10+	.22***	.34***	-.10+	.02	.19***	.03	-.24***	-.40***	-.23***	-.51***							
15 Positive Relations	3.81	0.77	.06	.02	.02	.53***	.42***	.38***	.51***	.23***	.04	-.43***	-.37***	-.18**	-.38***	.15**						
16 Personal Growth	4.18	0.51	.15**	.04	.01	.38***	.42***	.14**	.40***	.44***	.16**	-.23***	-.21***	-.14**	-.30***	.21***	.38***					
17 Purpose in Life	3.50	0.71	.19**	.23***	.16**	.57***	.65***	.26***	.45***	.40***	.13*	-.45***	-.35***	-.29***	-.33***	.24***	.37***	.45***				
18 Environmental Mastery	3.51	0.67	.01	.06	-.04	.68***	.69***	.29***	.41***	.27***	-.06	-.57***	-.54***	-.36***	-.54***	.38***	.52***	.44***	.66***			
19 Self-Acceptance	3.52	0.79	.01	.02	-.02	.75***	.78***	.34***	.51***	.31***	-.02	-.51***	-.50***	-.36***	-.54***	.35***	.58***	.43***	.66***	.82***		
20 Life Satisfaction	3.29	0.74	.08	.10+	.08	.67***	.62***	.42***	.52***	.20***	.02	-.47***	-.38***	-.28***	-.44***	.17**	.47***	.31***	.58***	.71***	.79***	

*Note*. Zero-order correlations are reported below the diagonal, partial correlations of admiration, adoration, and awe (controlling for the other two emotions) are reported above the diagonal.

***p < .001. **p < .01. *p < .05. + *p* < .10.

#### Admiration and adoration

We developed the Admiration and Adoration Scales (ADMADOS) to obtain a brief and face-valid measure. After extensive discussions within our interdisciplinary research group of the relevant literature (see Schindler et al. 2013) and personal accounts of admiration and adoration, we drafted an initial item set including six items reflecting admiration and seven items reflecting adoration. We pilot tested two preliminary versions of the questionnaire online with samples of 121 and 112 people. Based on item statistics and exploratory factor analyses, we developed a final reduced and revised set of eight items (Table 2).

**Table 2:** **The admiration and adoration scales (ADMADOS): items and factor loadings**

	**Factor loadings**	
**Item**	**USTD**	**STD**
*Admiration Scale*		
1 Things that someone accomplishes or has accomplished impress and elate me. / *Dinge, die jemand leistet oder geleistet hat, beeindrucken und begeistern mich.*	: 1	.73***
2 I admire someone for his/her characteristics or abilities. / *Ich bewundere jemanden für seine/ihre Eigenschaften oder Fähigkeiten.*	1.09***	.67***
3 I am continually impressed by something which someone does or has done. / *Ich bin nachhaltig beeindruckt von etwas, das jemand tut oder getan hat*.	1.30***	.80***
4 I feel that someone else’s ability or behavior is admirable. / *Ich empfinde das Können oder Handeln eines/einer anderen als bewunderungswürdig.*	1.02***	.69***
*Adoration Scale*		
1 I adore/worship an outstanding person or being. / *Ich verehre eine herausragende Figur.*	: 1	.77***
2 I perceive someone as an ideal representation of what is good and valued. / *Ich empfinde jemanden als Idealbild dessen, was gut und wertvoll ist.*	0.95***	.72***
3 I feel that I am shaped and guided by a special person or being. / *Ich spüre, wie ich durch eine besondere Figur geprägt und geleitet werde.*	0.93***	.76***
4 I perceive someone as such an outstanding person or being that he/she gives direction to my life. / *Ich empfinde jemanden als so herausragende Figur, dass er/sie für mein Leben richtungsweisend ist.*	1.16***	.87***

*Note*. USTD = unstandardized. STD = standardized. : 1 = set to 1.

***p < .001.

#### Joy and love

Items to measure joy and love were adapted from Trierweiler, Eid, and Lischetzke (2002). We formulated sentences including the four original terms for joy (e.g., “I am happy”), α = .90, and four terms for love (e.g., “I feel cared for by someone”), α = .86.

#### Pride

Our measure of pride was inspired by the 7-item Authentic Pride scale (Tracy and Robins 2007). To obtain an even briefer measure, we selected the three items of this scale that had obtained the highest loadings in Study 7 of Tracy and Robins (2007). We complemented this item set with one self-developed item asking directly about pride (“I am proud of myself”), resulting in four items total, α = .89.

#### Inspiration

We employed the Inspiration Scale (IS; Thrash and Elliot 2003) to assess the frequency of feeling inspired. This scale includes four items, α = .88.

#### Fascination and awe

As we did not find a published measure of fascination, we developed a brief fascination scale and refined it during pretesting. This measure was based on accounts of fascination (Kaplan 1995; Lüdtke et al. 2013) which have highlighted the potential to capture and hold a person’s attention as central feature of fascinating stimuli. The instrument consists of four items, α = .73, like “I feel an irresistible urge to closely attend to someone or something.”

We similarly developed a four-item measure of awe, α = .85. The only extant dispositional awe scale (Shiota, Keltner, and John 2006) conceptualizes awe rather broadly and with a focus on natural beauty as elicitor. We formulated items that would allow distinguishing awe from admiration and adoration and that tap into awe elicited by persons. In keeping with awe’s defining features (Keltner and Haidt 2003), the items focused on perceptions of vastness and need for accommodation, for instance, “I am overwhelmed and bewildered in view of the grandness of a person or object”.

#### Gratitude

We assessed dispositional gratitude with the Gratitude Questionnaire Six-Item Form (GQ-6; McCullough, Emmons, and Tsang 2002). Internal consistency of this scale was α = .73.

#### Envy

The Dispositional Envy Scale (DES; Smith, Parrott, Diener, Hoyle, and Kim 1999) was employed to assess individual differences in the tendency to feel envy. Participants rated how much they agreed with each of eight items, α = .84.

#### Sadness, fear, and shame

Participants responded to selected negative affect subscales of the Positive and Negative Affect Schedule – Expanded Form (PANAS-X; Watson and Clark 1994) by indicating how they feel in general. Sadness was assessed with five emotion adjectives, α = .86, and fear with six adjectives, α = .84. For theoretical reasons, I also analyzed the single PANAS-X item “ashamed”.

#### Psychological well-being

We assessed the six dimensions of PWB (Ryff 1989) with a 39-item short version of Ryff’s Scales of Psychological Well-Being (van Dierendonck 2005). The *autonomy* scale included eight items, α = .73, and the *personal growth* scale comprised seven items, α = .73. *Positive relations with others*, α = .80, *purpose in life*, α = .78, *environmental mastery*, α = .80, and *self-acceptance*, α = .89, were assessed with six items each.

#### Life satisfaction

We employed a German version (Glaesmer, Grande, Braehler, and Roth 2011) of the Satisfaction with Life Scale (SWLS; Diener, Emmons, Larsen, and Griffin 1985). This scale measures people’s satisfaction with their lives as a whole with five items, α = .82.

## Results

When analyzing the data, I first conducted a confirmatory factor analysis (CFA) of the ADMADOS. Second, the validity of the ADMADOS was determined by inspecting correlations and partial correlations of admiration and adoration with other emotions, personal growth, and purpose in life. Third, associations of admiration and adoration with other emotions and well-being were investigated in a multiple-step multiple mediator model. Prior to analyses, all variables were checked for univariate outliers and outlying scores were adjusted to the smallest or largest value that did not produce an outlier. Subsequently, multivariate outliers were identified as cases with Mahalanobis distance at *p* < .001 (Tabachnick and Fidell 1996). One participant emerged as multivariate outlier and was excluded from all analyses, leaving an *N* of 342.

### Factor structure of the admiration and adoration scales

The hypothesized CFA model with correlated admiration and adoration factors (Table 2) fit the data very well, χ^2^ (19) = 27.32, *p* = .10, *RMSEA* = .04, *CFI* = 0.99, *TLI* = 0.99. All items showed substantial loadings on their respective factor. The two-factor model also fit the data much better, Δχ^2^ (1) = 134.46, *p* < .001, than a one-factor model, χ^2^ (20) = 161.78, *p* < .001, *RMSEA* = .14, *CFI* = 0.88, *TLI* = 0.84.^b^ Although the latent admiration and adoration factors showed a substantial correlation of *r* = .74, *p* < .001 (the corresponding factor covariance is 0.37), they still were separable. For the following analyses, I used averaged admiration, α = .81, and adoration, α = .86, scores.

### Validating the admiration and adoration scales: relations with other emotions, personal growth, and purpose in life

Table 1 reports intercorrelations of all study variables. In line with Hypothesis 1, admiration and adoration were unrelated to joy and pride. In contrast, admiration and adoration showed significant positive correlations of small to medium size with love and gratitude (Hypothesis 2) as well as medium to large positive correlations with awe, inspiration, and fascination (Hypothesis 3).

The obtained correlations between the positive and negative emotions confirmed that admiration and adoration (as well as awe and fascination) are different from other positive emotions in that they also show positive or no relations with negative emotions. Correlations of joy, pride, love, gratitude, and inspiration with sadness, fear, shame, and envy all had negative signs and, with four exceptions, were significantly different from zero. In contrast, correlations of admiration, adoration, awe, and fascination with sadness, fear, shame, and envy were in the positive direction (with one exception) and sometimes were significantly different from zero (in line with Hypothesis 4). Specifically, admiration showed small positive correlations with fear and shame and a medium-sized positive correlation with envy. Contrary to Hypothesis 5, adoration was positively correlated with envy. Nevertheless, the association between adoration and envy, *r* = .11, was significantly smaller than the association between admiration and envy, *r* = .26, *z* = 3.28, *p* < .01.

Further in line with predictions, admiration showed a small positive correlation, *r* = .15, with personal growth (Hypothesis 6). This correlation was significantly greater than the nonsignificant correlation between adoration and personal growth, *r* = .04, *z* = 2.37, *p* < .05. Adoration was positively related to purpose in life, *r* = .23 (Hypothesis 7). However, this correlation was not significantly different from the correlation between admiration and purpose in life, *r* = .19, *z* = 0.88, *ns*.

As would be expected, admiration, adoration, and awe overlapped substantially. Therefore, I determined whether some of the reported associations were unique to admiration, adoration, or awe by computing partial correlations controlling for the other two emotions (e.g., partial correlations of admiration controlling for adoration and awe). As can be seen in Table 1 (above the diagonal), differences between admiration and adoration became more evident when looking at their unique associations. After controlling for adoration and awe, admiration showed a unique positive association with envy (Hypothesis 6). Adoration was negatively related to envy once admiration and awe were partialled out. Admiration demonstrated a unique positive association with personal growth (Hypothesis 7). Adoration demonstrated a unique positive association with purpose in life (Hypothesis 8).

### Admiration, adoration, and well-being: the mediating function of related emotions

Regarding Question 1, Table 1 reveals that admiration and adoration were unrelated to positive relations with others, environmental mastery, self-acceptance, and life satisfaction. Nevertheless, these findings leave questions on possible indirect associations of admiration and adoration with well-being constructs unanswered. The question whether admiration and adoration showed complex indirect associations with well-being variables as a result of their positive associations with other positive as well as negative emotions was of special interest in this regard (Question 2). Based on the obtained correlations, I selected four emotions for further study, namely, gratitude, inspiration, fascination, and envy. While gratitude and inspiration clearly demonstrated positive associations with well-being variables and envy clearly was related to poorer well-being, fascination was largely unrelated to well-being (Table 1). Fascination (rather than awe) was included in the model primarily for theoretical reasons, as it was expected to best capture the emotion underlying (a potentially unhealthy) obsession with a person or object.

In addition to gratitude, inspiration, fascination, and envy as mediators in a first step, the tested multiple-step multiple mediator model (Figure 1) included the six PWB dimensions as mediators in a second step, and life satisfaction as outcome variable. Mplus Version 6.1 was employed to run the analyses. I initially specified a model containing all possible paths from admiration and adoration to inspiration, fascination, gratitude, and envy, from these four emotions to the six PWB dimensions, and from the six PWB dimension to life satisfaction. Direct paths between admiration and adoration or the other four emotions and life satisfaction were not included, because I did not expect the emotions to have an additional effect on life satisfaction once the PWB dimensions were taken into account. Similarly, only two direct paths between admiration and adoration and the PWB dimensions were included based on theoretical considerations and the correlative evidence: the direct path between admiration and personal growth and the direct path between adoration and purpose in life. The decision to set direct paths to zero is supported by simulation studies which demonstrated that estimates of indirect effects are not affected by the magnitude of direct effects and, therefore, it is permissible to simplify the model in this way (MacKinnon, Lockwood, and Williams 2004). The covariance between admiration and adoration and the residual covariances between the mediators were estimated as part of the model. All predictor and mediator variables were centered at their means.

Although the hypothesized model fit the data quite well, χ^2^ (28) = 58.31, *p* < .001, *RMSEA* = .06, *CFI* = 0.99, *TLI* = 0.96, inspection of modification indices revealed that two additional paths should be included: the path between adoration and personal growth and the path between gratitude and life satisfaction. The resulting final model demonstrated a significant improvement in fit compared with the initial model, Δχ^2^ (2) = 28.38, *p* < .001, and a very good fit to the data, χ^2^ (26) = 29.93, *p* = .27, *RMSEA* = .02, *CFI* = 1.00, *TLI* = 0.99. This model is depicted in Figure 1, which includes the standardized path coefficients and the amount of variance explained (*R*^2^) in each mediator variable and life satisfaction.

As the primary aim of this analysis was to test for indirect effects of admiration and adoration on well-being variables, I obtained estimates of selected indirect effects. Indirect effects on purpose in life and personal growth were estimated because these two PWB dimensions were theoretically linked to admiration and adoration. In addition, the path model revealed that only self-acceptance and environmental mastery were positively related to life satisfaction when all six PWB dimensions were considered simultaneously. Therefore, indirect effects of admiration and adoration on self-acceptance and environmental mastery were investigated. Finally, I examined indirect effects of admiration and adoration on life satisfaction. Estimates of indirect and total effects are presented in Table 3. In addition to computing indirect effects, findings were corroborated by obtaining bias-corrected bootstrap confidence intervals (BC 95% CIs) of the indirect effects based on 5,000 bootstrap samples. The bias-corrected bootstrap has been recommended as the best method to establish indirect effects (MacKinnon et al. 2004; Preacher and Hayes 2008; Williams and MacKinnon 2008).

**Table 3:** **Mediated effects of admiration and adoration on well-being variables**

**Effect**	**Admiration**				**Adoration**			
	**Estimate**		**BC 95% CI**		**Estimate**		**BC 95% CI**	
	**USTD**	**STD**	**LL**	**UL**	**USTD**	**STD**	**LL**	**UL**
*Indirect Effects on Self-Acceptance (SA)*								
A → Inspiration → SA	0.05**	.05**	0.02	0.10	0.02+	.03+	0.00	0.05
A → Fascination → SA	−0.04*	-.03*	−0.08	−0.01	−0.04**	-.05**	−0.06	−0.01
A → Gratitude → SA	0.12***	.10***	0.05	0.19	0.03+	.04+	−0.01	0.07
A → Envy → SA	−0.14***	-.12***	−0.22	−0.08	0.03	.03	−0.01	0.06
*Total indirect:*								
A → Emotions^a^ → SA	−0.01	-.01	−0.13	0.12	0.04	.06	−0.03	0.11
*Effects on Purpose in Life (PL)*								
A → Inspiration → PL	0.07**	.06**	0.02	0.12	0.03*	.04*	0.00	0.06
A → Fascination → PL	−0.02	-.02	−0.07	0.01	−0.02	-.03	−0.05	0.01
A → Gratitude → PL	0.08***	.08***	0.04	0.15	0.02	.03	−0.00	0.05
A → Envy → PL	−0.07**	-.06**	−0.12	−0.03	0.01	.02	−0.00	0.03
*Total indirect:*								
A → Emotions^a^ → PL	0.06	.06	−0.03	0.16	0.04	.06	−0.01	0.10
*Total indirect + direct:*								
A → PL	—	—	—	—	0.12**	.18**	0.05	0.19
*Effects on Personal Growth (PG)*								
A → Inspiration → PG	0.06**	.08**	0.02	0.11	0.02*	.05*	0.00	0.05
A → Fascination → PG	0.01	.01	−0.01	0.03	0.01	.01	−0.01	0.03
A → Gratitude → PG	0.05**	.07**	0.02	0.09	0.01	.03	−0.00	0.04
A → Envy → PG	−0.05**	-.06**	−0.09	−0.02	0.01	.02	−0.00	0.02
*Total indirect:*								
A → Emotions^a^ → PG	0.07*	.09*	−0.00	0.14	0.05*	.10*	0.01	0.09
*Total indirect + direct:*								
A → PG	0.16**	.21**	0.05	0.26	−0.05	-.09	−0.11	0.02
*Indirect Effects on Environmental Mastery (EM)*								
A → Inspiration → EM	0.04**	.04**	0.02	0.08	0.02+	.03+	0.00	0.04
A → Fascination → EM	−0.03*	-.03*	−0.08	−0.01	−0.03**	-.05**	−0.06	−0.01
A → Gratitude → EM	0.07***	.07***	0.03	0.12	0.02	.03	−0.00	0.05
A → Envy → EM	−0.13***	-.13***	−0.20	−0.07	0.02	.04	−0.01	0.06
*Total indirect:*								
A → Emotions^a^ → EM	−0.05	-.05	−0.14	0.05	0.03	.05	−0.03	0.09
*Indirect Effects on Life Satisfaction (LS)*								
A → Inspiration → PWB^b^ → LS	0.03*	.03*	0.01	0.06	0.01+	.02+	0.00	0.03
A → Fascination → PWB^b^ → LS	−0.03**	-.03**	−0.07	−0.01	−0.03**	-.04**	−0.06	−0.02
A → Gratitude → PWB^b^ → LS	0.08***	.07***	0.04	0.14	0.02	.03	−0.00	0.05
A → Envy → PWB^b^ → LS	−0.08***	-.07***	−0.14	−0.04	0.02	.02	−0.01	0.04
*Total indirect* ^c^ *:*								
A → LS	0.03	.03	−0.07	0.14	0.05	.07	−0.01	0.11

*Note*. USTD = unstandardized. STD = standardized. BC 95% CI = bias-corrected 95% confidence interval for unstandardized estimate based on 5,000 bootstrap samples. LL = lower limit. UL = upper limit. A = admiration or adoration. — = total effect not reported (model does not include direct path).

^a^Emotions includes the combined indirect effects via inspiration, fascination, gratitude, and envy.

^b^PWB = psychological well-being, which includes the combined indirect effects via positive relations, self-acceptance, purpose in life, personal growth, environmental mastery, and autonomy.

^c^Total indirect effect includes all possible two-step and three-step mediated paths between admiration or adoration and life satisfaction depicted in Figure 1.

***p < .001. **p < .01. *p < .05. + *p* < .10.

Table 3 shows that admiration and adoration had significant indirect effects on self-acceptance, purpose in life, personal growth, environmental mastery, and life satisfaction. Specifically, admiration was associated with greater inspiration and gratitude and, by way of these associations, had positive indirect effects on self-acceptance, purpose in life, personal growth, environmental mastery, and life satisfaction. However, admiration also was related to greater fascination and envy, and these associations provided for significant negative indirect effects on self-acceptance, environmental mastery, and life satisfaction. Envy, but not fascination, further served as mediator of negative associations of admiration with purpose in life and personal growth. Admiration thus had simultaneous positive and negative indirect effects on well-being variables. It should be noted that all significant effects reported in Table 3 represent small effects. For instance, by way of admiration’s indirect pathway through envy, an increase in admiration by one point on the 5-point scale would lead to a decline in self-acceptance by 0.14, which amounts to *d* = 0.18.

Table 3 further reveals that when the significant indirect effects through inspiration, fascination, gratitude, and envy were combined (see reported total indirect effects), they cancelled each other out, resulting in a nonsignificant overall effect of admiration on well-being. The PWB dimension personal growth was the only exception from this general pattern. Admiration showed a significant total effect of *B* = 0.16 on personal growth.

Findings for adoration were less pronounced than those for admiration. After controlling for admiration, adoration was significantly positively related only to inspiration and fascination, but unrelated to gratitude and envy. Therefore, only some positive indirect effects of adoration through inspiration and some negative indirect effects of adoration through fascination were obtained. By way of its association with inspiration, adoration had a positive impact on purpose in life and personal growth. It should be noted that the initially only marginally significant indirect effects of adoration through inspiration on self-acceptance, environmental mastery, and life satisfaction all were significant at *p* < .05 in the bootstrap analysis (see BC 95% CIs in Table 3). By way of its association with fascination, adoration had a negative effect on self-acceptance, environmental mastery, and life satisfaction. Adoration did not show significant total indirect effects on well-being variables, with one exception: Adoration had a significant total effect on purpose in life, *B* = 0.12, which was primarily attributable to the significant direct path from adoration to purpose in life.

## Discussion

In light of (self-help) publications suggesting that people benefit from admiration and adoration (Gallozzi 2009; Perticone 2007; Rattner and Danzer 2006; Woodruff 2001), it was timely to examine whether these emotions should be considered in well-being research. Are they linked to greater well-being? If one conceptualizes well-being as subjective well-being, the answer provided by the present data is no. If, however, one takes a broader perspective on what constitutes well-being, the answer may well be yes.

### Measuring admiration and adoration: reliability and validity

As a first step towards understanding admiration and adoration, it was necessary to develop an instrument assessing the disposition to experience the two emotions. The resulting ADMADOS have performed very well in this study. They showed the expected two-factorial structure and high internal consistencies.

Admiration and adoration demonstrated the hypothesized pattern of correlations with other emotions. They were unrelated to joy and pride as positive emotions which typically result from own goal progress or need fulfillment (Hypothesis 1). Admiration and adoration were positively related to other emotions expressive of the disposition to appreciate others, namely, love and gratitude (Hypothesis 2). Admiration and adoration further were linked to other emotions reflective of a disposition to respond positively to stimuli that challenge one’s knowledge and understanding, namely, awe, inspiration, and fascination (Hypothesis 3).

When looking at associations with sadness, fear, shame, and envy, admiration and adoration (along with awe and fascination) turned out to be rather atypical positive emotions. In contrast to other positive emotions, admiration, adoration, awe, and fascination were unrelated or even positively related to negative emotions (Hypothesis 4). The findings further confirmed a unique positive association between dispositional envy and admiration. Although adoration showed a small zero-order positive correlation with envy, this association became negative after controlling for admiration and awe (Hypothesis 5). In contrast to adoration, both admiration and envy are emotions that can occur in upward social comparison situations and reflect a desire to move closer to an ideal state seen in the other (cf. Cohen-Charash 2009; Smith 2000; van de Ven et al. 2011). The positive relation between the two emotions supports the prediction that what people highly value can cause positive but also negative emotions when obtained by others. When averaging across situations relevant to an ideal, the ideal’s importance should determine the intensity of the emotional response but not its valence, thus giving rise to a positive correlation between admiration and envy.

While it was difficult to predict how admiration and adoration relate to different well-being indicators, two predictions could be derived from a theoretical analysis of the two emotions (Schindler et al. 2013). As expected, admiration was positively related to personal growth (Hypothesis 6) and adoration showed a positive relation with purpose in life (Hypothesis 7). Inspection of partial correlations confirmed that the relation with personal growth was unique to admiration. Purpose in life showed a unique positive association with adoration.

### Relations of admiration and adoration with well-being: the role of related emotions

The first open research question (Question 1) concerned relationships of admiration and adoration with dimensions of PWB (other than personal growth and purpose in life) and life satisfaction. Nonsignificant correlations of admiration and adoration with positive relations with others, environmental mastery, self-acceptance, and life satisfaction suggested that the two emotions are not relevant to well-being. This matches well with the finding that the conceptually related character strength to appreciate beauty and excellence shows very small or nonsignificant associations with life satisfaction (Peterson, Ruch, Beermann, Park, and Seligman 2007). The finding is somewhat at odds with recent evidence suggesting that awe, another emotion that is closely related to this character strength and also to adoration, can help increase life satisfaction (Rudd et al. 2012). However, the present findings also did not support this beneficial role of awe. Our measure of awe was unrelated to life satisfaction and all PWB dimensions except for purpose in life. Thus, it is likely that awe experienced in response to panoramic views and natural beauty (as in Rudd et al. 2012) and in response to superior others has different well-being consequences.

The findings for admiration and adoration are not too surprising if we consider that these emotions help generate and increase commitment to ideals and values (Schindler et al. 2013), which are highly abstract goals that can never be completely attained. Goal systems are characterized by a trade-off between meaning and manageability (Little 1989; McGregor and Little 1998): the most meaningful goals are the ones that are least likely to be attained and to, thereby, increase life satisfaction. Thus, admiration and adoration may indicate the presence of meaningful goals, but not of the means for goal attainment. They do not motivate the individual to do what he or she readily is capable of doing and, thereby, enable immediate goal progress (see van de Ven et al. 2011, for relevant findings on admiration), but rather encourage people to embark on a long-term journey that may be fraught with difficulties and frustrations.

The second open research question (Question 2) asked whether taking the other positive and negative emotions associated with admiration and adoration into account helps elucidate the relationship between the two emotions and well-being. Significant but counteractive mediated paths can be hidden beneath an overall nonsignificant association between predictor and outcome (e.g., Hayes 2009; Preacher and Hayes 2008). I assumed that the effects of admiration and adoration on well-being can, in part, be explained by considering associated positive and negative emotions. A multiple-step multiple mediator model revealed positive indirect effects of admiration and adoration on well-being indicators through inspiration and gratitude (only for admiration).

Inspiration includes two components that explain its role as a mediator of this relationship: being *inspired by* and being *inspired to* (Thrash and Elliot 2004; Thrash, Elliot, Maruskin, and Cassidy 2010). The inspired-by component shows considerable overlap with adoration and can be considered as an instance of admiration (cf. Schindler et al. 2013; Thrash and Elliot 2004). This component reflects the inspiring stimulus’s intrinsic value as something that evokes accommodation of knowledge structures and transcendence of prior concerns. The inspired-to component provides a link to well-being constructs, because it reflects approach motivation to actualize the newly apprehended possibilities. It is this motivation to action, which does not necessarily occur in the wake of admiration or adoration, that explains how admiration and adoration can contribute to self-improvement, greater purpose in life, improved environmental mastery, and life satisfaction.

In addition to inspiration, gratitude emerged as a mediator of positive effects of admiration on well-being. Although gratitude is connected with inspiration (people are grateful toward sources of inspiration; Thrash et al. 2010), there were unique indirect effects of admiration via gratitude on well-being. This underscores that gratitude does more to enhance well-being than to ready individuals to appreciate others for providing new insights and energy for goal striving (cf. Emmons and Mishra 2011; Fredrickson 2004; McCullough et al. 2001; Watkins 2004). What distinguishes gratitude from inspiration is the recognition of provisions from others as gifts. That is, these favors have been intentionally given and express the benefactor’s valuation of the beneficiary (cf. McCullough et al. 2001; Watkins 2004). Gratitude thus includes a social aspect that is not part of inspiration: it helps develop and strengthen social bonds (Emmons and Mishra 2011; Fredrickson 2004). The resulting positive relationships, together with other outcomes that have been associated with gratitude such as better coping with adversity and increased motivation for moral behavior (Emmons and Mishra 2011; Fredrickson 2004; Watkins 2004), account for gratitude’s strong link to most PWB dimensions (except for autonomy) and life satisfaction.

In addition to positive indirect effects, the mediator model also revealed negative indirect effects of admiration and adoration through fascination and envy (only for admiration) on well-being. Fascination showed complex associations with well-being. Fascination was mostly unrelated to PWB dimensions and life satisfaction when considering zero-order correlations but showed significant negative associations with self-acceptance and environmental mastery once other emotions were included as rival predictors. Consequently, the indirect effects of both admiration and adoration via fascination on self-acceptance, environmental mastery, and life satisfaction were negative. On the one hand, the occurrence of fascination together with admiration and adoration may be functional. Fascination disrupts ongoing goal pursuit and makes the individual pay attention to something that is not of immediate concern to him or her. It may sometimes take a disruptive experience to awaken people to new possibilities (similar arguments have been put forward for awe, Keltner and Haidt 2003, and inspiration, Thrash and Elliot 2004).

On the other hand, fascination can be evoked by stimuli that do not offer the potential for growth or self-improvement. This has been highlighted in the literature on celebrity worship, which proposes that people can become so obsessed with a celebrity that they neglect other important aspects of their lives (cf. Maltby and Giles 2008). Taken together, this suggests that the unique indirect effects of admiration/adoration via fascination (after controlling for its overlap with inspiration) on well-being can be attributed to the obsessive element included in fascination, which undermines mastery of competing tasks and induces negative evaluations of oneself.

The positive connection between admiration and envy gave rise to negative indirect effects of admiration on all PWB dimensions and life satisfaction. As people who are prone to experience admiration also are prone to experience envy, it may well be that envious feelings are the price one has to pay for frequent experiences of admiration.

In sum, the mediator model uncovered that admiration and adoration were unrelated to well-being because these emotions had positive and negative indirect effects through related emotions on well-being indicators that cancelled each other out. The resulting total effects on self-acceptance, environmental mastery, and life satisfaction were nonsignificant, while small total effects of admiration on personal growth and of adoration on purpose in life were retained.

### Limitations and future research directions

There are some limitations of this study to be acknowledged. First, this study was conducted in Germany and, therefore, we do not know whether the present findings would generalize to other countries and cultures. An important consideration is that the German language offers only one term (*Verehrung*) to translate adoration, reverence, worship, and veneration. This term, in contrast to admiration (*Bewunderung* in German), can have a negative flavor to it, evoking associations of being deluded and brain-washed. Therefore, it is well possible that experiences of adoration were underreported in this study, although we made every effort to word the adoration items in a way that would not evoke negative associations.

Second, the current analyses did not differentiate between forms of admiration or adoration depending on the ideals and values that elicit the emotion. Algoe and Haidt (2009) have demonstrated interesting differences between admiration for virtue (i.e., moral elevation) and admiration for skill. Therefore, it is possible that admiration for a specific ideal (e.g., virtue) may link to subjective well-being while admiration for other ideals (e.g., skill) does not. It may also be the case that the effects of admiration and adoration depend on who elicits the respective emotion. It thus would be an interesting direction for future research to determine whether associations of admiration and adoration with well-being indicators vary as a function of the embodied ideals or the emotion’s target person.

Third, this paper only investigated potential individual benefits of admiration and adoration. However, especially adoration theoretically should function to bind communities together (see Schindler et al. 2013). Thus, adoration can be expected to relate to measures of collectivistic orientations (cf. Triandis and Gelfand 1998) or values with a social focus (such as benevolence or conformity; cf. Schwartz et al. 2012). We actually have included a measure of collectivistic orientations (Sivadas, Bruvold, and Nelson 2008) in this study and found a partial (controlling for admiration) correlation of *r* = .23, *p* < .001, between adoration and vertical collectivism (i.e., an emphasis on hierarchy and sacrificing one’s self-interest for the group). In contrast, admiration showed a partial (controlling for adoration) correlation of *r* = .12, *p* < .05, with horizontal collectivism (i.e., an emphasis on equality and willingness to cooperate without submitting to others). After controlling for the respective other emotion, adoration was not significantly related to horizontal collectivism and admiration was not significantly related to vertical collectivism. As, however, the employed scales had low internal consistencies, it would take future research to corroborate such associations of admiration and adoration with collectivism.

Fourth, the finding that admiration and adoration are unrelated to pride probably is limited to pride in oneself. If we had assessed pride in someone else in addition to pride in oneself, we would have expected to see positive correlations of admiration and adoration with pride in others.

## Conclusions

Some important conclusions on admiration and adoration can be drawn from this study. For the person who seeks to increase his or her life satisfaction, advice to admire and adore is misguided. This finding is noteworthy in light of the strong evidence for associations between gratitude and well-being indicators (cf. Emmons and Mishra 2011; Watkins 2004), which once more were confirmed in this study, and the usefulness of gratitude interventions for increasing well-being (cf. Peterson and Seligman 2004). As admiration, adoration, and gratitude all belong to the other-praising emotion family (Algoe and Haidt 2009; Haidt 2003b), it might be assumed that admiration and adoration also have effects similar to those of gratitude and could equally well be employed in interventions. The present findings suggest that this would not work and rather provide support for the unique potential of gratitude to foster life satisfaction.

Nevertheless, admiration and adoration are relevant to well-being when we employ a broader understanding of well-being as eudaimonic well-being that encompasses dimensions of PWB. Although people who experience personal growth or who have a sense of purpose in life are not necessarily the happiest ones, growth and purpose are considered to be ingredients of a good life (cf. Delle Fave et al. 2011; King and Napa 1998; McGregor and Little 1998; Vittersø and Søholt 2011). The present findings revealed that admiration was linked to growth while adoration was linked to purpose. These emotions seem to bind people to ideals regardless of their ability to move closer to them. Thus, the primary function of admiration and adoration may lie in the promotion of collective rather than individual well-being by fostering cooperation and prosocial values. For instance, when viewed at the societal level, admiration is an emotion that alerts people to outgroups whose cooperation should be sought (e.g., Caprariello, Cuddy, and Fiske 2009). Moreover, in advancing the notion of *sustainable well-being*, Kjell (2011) highlighted the interdependencies of individuals with others and nature. As individual well-being can be sustained at the cost of others, the functioning of a community depends on mechanisms that constrain self-interest and increase collective well-being. It should be fruitful to focus on indicators of collective rather than individual well-being in future research on potential benefits of admiration and adoration.

## Endnotes

^a^The present study was conducted within a larger research group on adoration and admiration with this paper’s author as principal investigator. As other members of this research group were involved in planning and conducting the study (see acknowledgements), I use the personal pronoun “we” when presenting the study to honor their involvement and support. Throughout the paper, I nevertheless use the personal pronoun “I” when referring to my own perspective as this paper’s author.

^b^The factorial structure of the ADMADOS was confirmed in another sample (*N* = 242; predominantly university students; 65.3% women; age range 18–64 years, *M* = 27.0 years). The two-factor model with correlated admiration and adoration factors showed an acceptable fit to the data, χ^2^ (19) = 38.00, *p* < .01, *RMSEA* = .06, *CFI* = 0.98, *TLI* = 0.96, which was vastly superior, Δχ^2^ (1) = 188.54, *p* < .001, to the fit of an alternative one-factor model, χ^2^ (20) = 226.54, *p* < .001, *RMSEA* = .21, *CFI* = 0.73, *TLI* = 0.63. All items had significant loadings on their respective factor (standardized loadings between .66 and .86). The admiration, α = .83, and adoration, α = .83, scales had good reliabilities.
